# Comparative Transcriptome Analyses Indicate Molecular Homology of Zebrafish Swimbladder and Mammalian Lung

**DOI:** 10.1371/journal.pone.0024019

**Published:** 2011-08-26

**Authors:** Weiling Zheng, Zhengyuan Wang, John E. Collins, Robert M. Andrews, Derek Stemple, Zhiyuan Gong

**Affiliations:** 1 Department of Biological Sciences, National University of Singapore, Singapore, Singapore; 2 Vertebrate Development and Genetics, Wellcome Trust Genome Campus, Wellcome Trust Sanger Institute, Hinxton, Cambridge, United Kingdom; Auburn University, United States of America

## Abstract

The fish swimbladder is a unique organ in vertebrate evolution and it functions for regulating buoyancy in most teleost species. It has long been postulated as a homolog of the tetrapod lung, but the molecular evidence is scarce. In order to understand the molecular function of swimbladder as well as its relationship with lungs in tetrapods, transcriptomic analyses of zebrafish swimbladder were carried out by RNA-seq. Gene ontology classification showed that genes in cytoskeleton and endoplasmic reticulum were enriched in the swimbladder. Further analyses depicted gene sets and pathways closely related to cytoskeleton constitution and regulation, cell adhesion, and extracellular matrix. Several prominent transcription factor genes in the swimbladder including *hoxc4a*, *hoxc6a*, *hoxc8a* and *foxf1* were identified and their expressions in developing swimbladder during embryogenesis were confirmed. By comparison of enriched transcripts in the swimbladder with those in human and mouse lungs, we established the resemblance of transcriptome of the zebrafish swimbladder and mammalian lungs. Based on the transcriptomic data of zebrafish swimbladder, the predominant functions of swimbladder are in its epithelial and muscular tissues. Our comparative analyses also provide molecular evidence of the relatedness of the fish swimbladder and mammalian lung.

## Introduction

The swimbladder is a specialized organ in teleosts that regulates buoyancy. It is a sac filled by several types of gas, mainly oxygen and carbon dioxide [1], [2], and is located between the peritoneum and the vertebral column in the dorsal part of the body. The volume of gas in the swimbladder can be actively regulated to maintain neutral buoyancy as fish ascend or descend in the water column. The long-term maintenance of internal gas pressure and also compensatory inflation and deflation are under reflex autonomic control. The homology of the vertebrate lung and swimbladder was noted by the British comparative anatomist Richard Owen as early as in 1846 [3]. It has been noted that both the swimbladder and lung are originated from the same ancestral organ, namely the respiratory pharynx, which is the posterior region of the pharynx [4], [5]. The diversified morphologies and functions of the swimbladder in different fish species illustrate its evolutionary relationship with tetrapod lungs. All ray-finned fish except the Polypteriformes develop the dorsal part directly from the ancestral respiratory pharynx as a pulmonoid swimbladder, which has a homologous blood supply with the lung. Although the homology of the lung and swimbladder has been well recognized based on morphological and embryological evidence, molecular evidence is still lacking [6], [7].

Despite the publication of a few papers recently on zebrafish swimbladder development, the swimbladder is still an organ understudied [8], [9], [10], [11]. In particular, we have characterized in detail the early development of zebrafish swimbladder with three distinct tissue layers [9]. Our study has also illustrated some conserved gene expression and regulatory mechanisms during early swimbladder and lung development, including the Hedgehog signaling pathway [9]. The study provides evidence that the budding and initial growth of the two organs is conserved, and that the Hedgehog signaling pathway is involved in the early development of the two organs. Thus, the difference of the two organs is likely to lie in the branching morphogenesis in lung, which is absent in the swimbladder.

Transcriptomic analyses, both descriptive and quantitative, are important for interpreting the functional elements of the genome and revealing the molecular constituents of cells and tissues. The transcriptome of zebrafish tissues have been characterized based on expressed sequence tag (EST) or microarray techniques [12], [13], [14], [15]. With the rapid advance of DNA sequencing technology, here we used Illumina next generation sequencing (NGS) platform for high content analysis of the zebrafish swimbladder transcriptome. We first described the molecular constitution of this organ, and then focused on the unique features, including the enriched genes, transcription factors and biological pathways. We also established the relatedness between fish swimbladder and mammalian lung by transcriptome comparison.

## Results

### General features of the zebrafish swimbladder transcriptome

**Figure 1:**
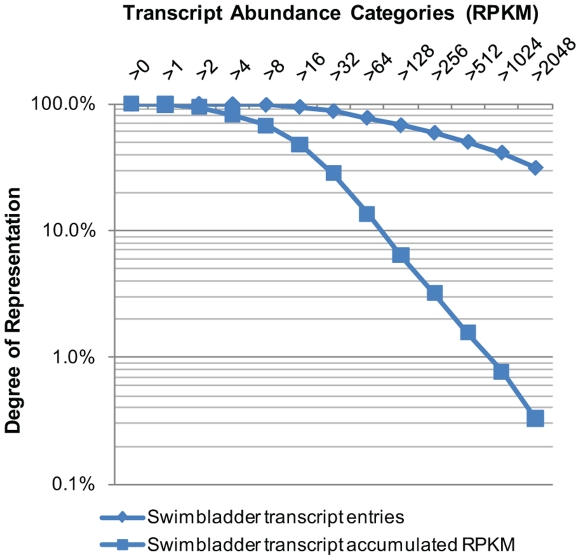
Distribution of transcript entries and total transcript counts over different tag abundance categories. Categories of transcript abundance were assigned by setting the lower limit of the count number that includes the transcript as a category member. The percentages of total transcript counts and number of different transcript entries per category are plotted on a logarithmic scale (base 10).

**Figure 2:**
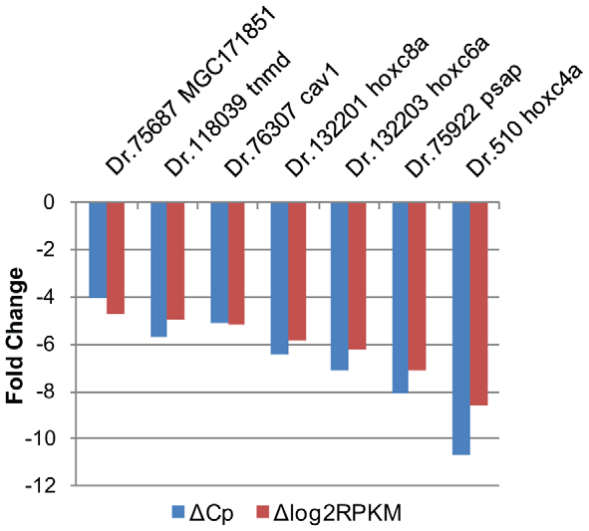
Real-time validation of RNA-seq data. The relative expression level of the genes selected was shown in log_2_ fold change as compared with a housekeeping gene, *ef1a* (10680.1 RKPM).

The swimbladders were isolated from 90 adult zebrafish and pooled to make representatives for deep sequencing analysis. One cDNA library was constructed and sequenced for the swimbladder. A total of 34 million of read pairs was generated (Table S1), which is comparable to several recently published data using the Illumina Genome Analyzer [16], [17]. All sequence tags were mapped to known transcripts in ZGC (Zebrafish Gene Collection) in order to reveal the molecular characteristics of the swimbladder transcriptome. A total of 9,315 transcript entries were identified with as few as one mapped read pairs, constituting 55.6% of total known zebrafish transcript entries in the ZGC database. As indicated in Figure 1, the swimbladder transcriptome showed a relatively continuous distribution of gene expression levels. Similar to previous RNA-seq studies in other tissues [17], there were only a few transcripts which had high expression levels, while most transcripts were expressed at very low levels. More than 60% of the transcript body consisted of the highest expressed transcripts which accounted for less than 10% of the transcript entries, while the lowest expressed 60% of the transcript entries only contributed for 10% of the total transcript counts. It has been documented in previous studies that RNA-seq can readily detect gene expression level across a broad dynamic range [18], [19]. The expression level of genes in the swimbladders ranged from 0.54 to 11,178 RPKM, showing a dynamic range of more than five orders of magnitude in RNA concentration. Real-time PCR was carried out to verify relative abundance of several selected transcripts determined by RNA-seq and the result indicated a good correlation of the two methods (Figure 2). Since transcripts with marginal expression levels could be due to leaky expression, we implemented a general cutoff at 10 RPKM (∼3 transcripts per cell) for analyzing physiologically more relevant transcripts [18]. Finally, 5,758 transcript entries above the cutoff were used to represent the total swimbladder transcriptome. The list was subsequently mapped to 5,506 zebrafish Unigene clusters.

### Functional implications of the swimbladder transcriptome

**Figure 3:**
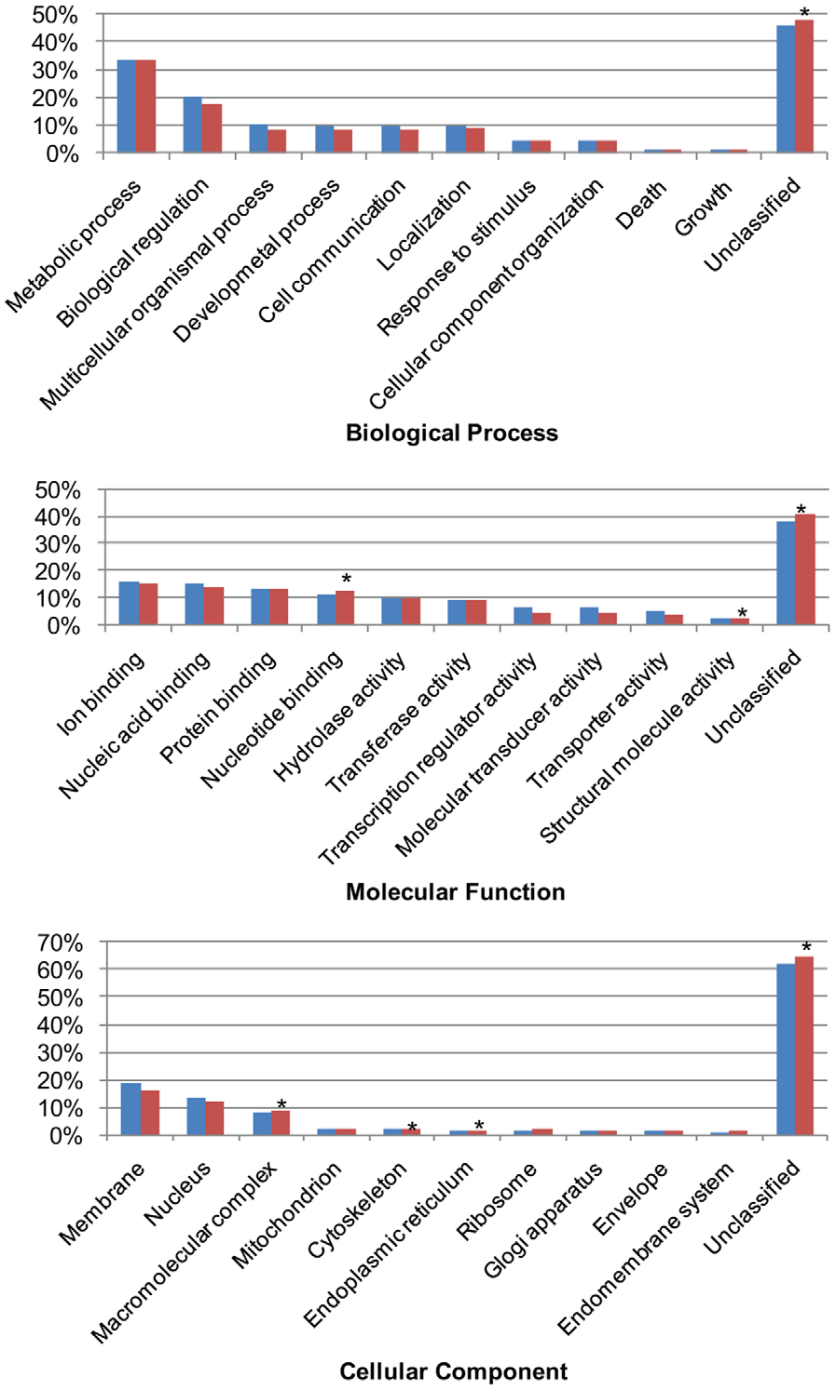
Gene ontology slim classification for the entire swimbladder transcriptome under Biological process, Molecular function and Cellular component classifications. Slim classifications of the total ZGC database entries and swimbladder transcriptome are represented by blue and red bars, respectively. Astrid is used to label significantly enriched categories in the swimbladder (FDR<0.01).

The 5,506 zebrafish Unigene clusters identified in the swimbladder were classified based on Gene Ontology (GO). Comparing to the distribution of GO categories of the total ZGC database (9,631 Unigene clusters), the swimbladder had significantly more expressed genes with unknown function in all the three classifications: Biological Process, Molecular Function, and Cellular Component, indicating the fact that the swimbladder is a less studied organ (Figure 3, Table S2). Under the Biological Process classifications, large proportions of genes were involved in housekeeping functions such as metabolic process and biological regulation. In Molecular Function classification, the categories of nucleotide binding and structural molecular activity were significantly enriched in the swimbladder, whereas in Cellular Component classification genes functioning in the endoplasmic reticulum was enriched in the swimbladder, suggesting the active synthesis and transportation of proteins. In particular, enriched categories under Molecular Function and Cellular Component together implicated the abundance of cytoskeleton genes in the swimbladder.

**Figure 4:**
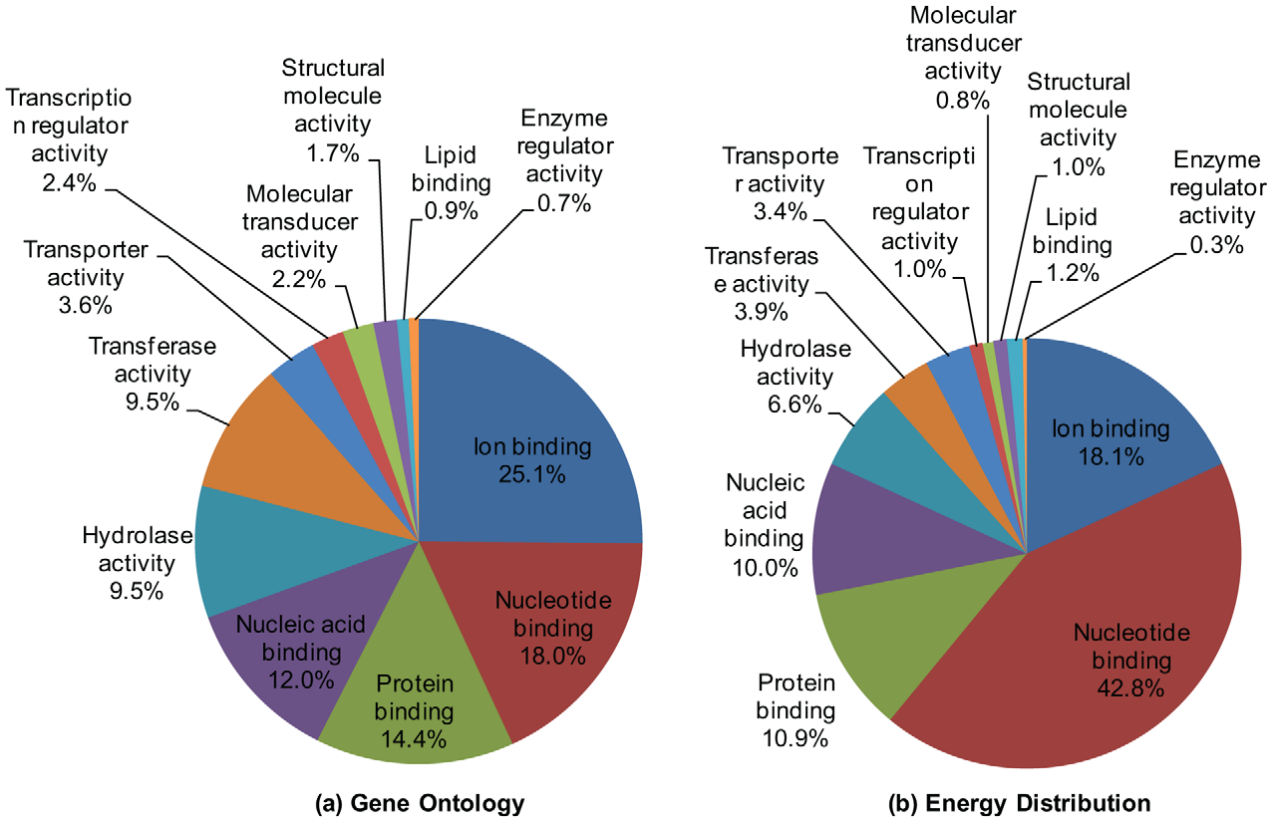
Gene ontology classification (a) and energy distribution (b) of the swimbladder transcriptome. Gene ontology classification and energy distribution are based on GO Slim classification of molecular functions. Genes without gene ontology information constitute 40% of the total swimbladder transcriptome and they were not included in the pie chart.

Next we compared the Gene Ontology and energy distribution of the swimbladder under Molecular Function category. Energy distribution describes how a given tissue distributes its transcriptional energy based on relative abundance of total transcripts in different GO groups, thus yielding information on the main function of the tissue [13]. As shown in Figure 4, genes with nucleotide binding function are the second most diversified group in the swimbladder, and this group occupies a much heavier proportion in the energy distribution, indicating these transcripts tend to have higher expression levels. At the same time, there were a few categories which showed high diversity but have low expression levels, including genes with hydrolase, transferase, transcription regulator, molecular transducer, and enzyme regulator activities. These categories are crucial for maintaining basic metabolisms and performing specific functions for the swimbladder, although they are expressed at relatively lower levels.

The original swimbladder transcriptome list contains many ribosomal protein genes and other housekeeping genes (see Table S3). To extract a more specific swimbladder transcriptome, a list of 888 enriched genes in the swimbladder was generated (bold-labeled in Table S3) using t-test by comparing with three other sets of zebrafish transcriptome data from the heart, brain and head kidney. The list efficiently excluded commonly expressed housekeeping genes and retained rarely expressed genes coding for transcription factor and signaling activity if they were enriched in the swimbladder.

A detailed enrichment analysis of GO terms was performed to examine the functional distribution of the 888 enriched genes (Table S4). Genes located in the endoplastic reticulum and extracellular region were enriched in the list, which implicated the active protein modification and transportation in the swimbladder. The results of enriched functional group in terms of Biological Process and Molecular Function together support the enrichment of signaling molecules in the list. We furthered examined the composition of these signaling molecules (Table S5). Among the 201 zebrafish Unigenes identified in the KEGG pathway database, 31 of them were involved in focal adhesion or extracellular matrix (ECM)-receptor interaction, suggesting the critical role of ECM in the swimbladder. Genes involved in adherens junction and tight junction were also enriched, which is essential for epithelial morphology and function. Particularly, genes involved in Hedgehog and TGF beta signaling pathways were enriched. Previous research in the lab has shown that Hedgehog signaling is critical for swimbladder specification and organization during embryogenesis [9]. The current transcriptome data correlates with the early developmental mechanism, suggesting that Hedgehog pathway remains active in the adulthood stage and may be important to maintain swimbladder regular function. Furthermore, GSEA (gene set enrichment analysis) pre-ranked analysis produced similar results in a quantitative manner (Table S6).

### Top enriched genes in the swimbladder

In the list of top 50 transcribed genes (Table 1), the most abundant category was extracellular matrix (13 zebrafish Unigenes). Among them, three different glycoprotein genes were present: *sparc*, *dcn* and *chad*.

**Table 1:** Top 50 enriched Unigenes in the swimbladder with annotation.

	UGCluster	Gene Symbol	Gene Name	RPKM	P value
1	Dr.20277	acta2	Actin, alpha 2, smooth muscle, aorta	75742.5	1.17E-06
2	Dr.82256	tagln	Transgelin	7296.7	2.43E-05
3	Dr.75554	sparc	Secreted acidic cysteine rich glycoprotein	3751.4	3.04E-06
4	Dr.75575	col1a2	Collagen, type I, alpha 2	2351.1	1.94E-04
5	Dr.76397	mmp2	Matrix metalloproteinase 2	2038.5	6.96E-05
6	Dr.105413	tpm1	Tropomyosin 1 (alpha)	1903.5	1.90E-05
7	Dr.76093	s100a10b	S100 calcium binding protein A10b	1821.1	8.08E-03
8	Dr.67796	icn	Ictacalcin	1584.0	4.87E-03
9	Dr.42794	ctgf	Connective tissue growth factor	1537.3	6.25E-05
10	Dr.24504	pabpc1a	Poly A binding protein, cytoplasmic 1 a	1248.1	1.14E-03
11	Dr.114623	cald1	Caldesmon 1	1226.2	5.84E-04
12	Dr.79279	lum	Lumican	1175.7	3.22E-05
13	Dr.34240	fbp2	Fructose-1,6-bisphosphatase 2	1053.9	3.96E-06
14	Dr.79127	stm	Starmaker	1023.5	9.47E-03
15	Dr.89765	zgc:103467	Myosin, light chain 9, regulatory	1014.0	1.90E-03
16	Dr.122523	dap1b	Death associated protein 1b	944.6	1.09E-02
17	Dr.76351	dcn	Decorin	839.7	2.12E-04
18	Dr.77427	b2m	Beta-2 microglobulin	798.2	1.41E-04
19	Dr.7877	mylka	Myosin, light chain kinase a	718.8	3.27E-04
20	Dr.76950	tpm4	Topomyosin alpha-4 chain	699.1	7.02E-04
21	Dr.88679	fhl2a	Four and a half LIM domains 2a	693.6	5.34E-03
22	Dr.43046	c1qtnf1	C1q and tumor necrosis factor related protein 1	682.7	1.05E-06
23	Dr.155448	bactin2	Bactin2	661.0	2.56E-04
24	Dr.86222	rergl	RERG/RAS-like	651.3	1.82E-04
25	Dr.80811	si:ch211-237l4.5	Si:ch211-237l4.5	628.6	2.24E-03
26	Dr.80402	chad	Chondroadherin	558.6	5.77E-06
27	Dr.142266	itgb1b	Integrin, beta 1b	536.1	7.00E-03
28	Dr.105090	ckba	Creatine kinase, brain a	480.9	3.89E-03
29	Dr.110680	mxra8a	Matrix-remodelling associated 8a	476.5	4.97E-05
30	Dr.150732	sdc2	Syndecan 2	465.2	7.39E-04
31	Dr.104772	LOC100332090	Thymosin, beta 4-like	453.5	1.15E-02
32	Dr.78058	myh11	Myosin, heavy polypeptide 11	448.5	2.10E-03
33	Dr.33255	tpm3	Tropomyosin 3	428.3	1.37E-02
34	Dr.81804	dkk3	Dickkopf homolog 3 (Xenopus laevis)	427.7	1.86E-04
35	Dr.76952	actn4	Actinin, alpha 4	407.0	2.55E-03
36	Dr.29018	cygb1	Cytoglobin 1	391.4	6.09E-05
37	Dr.30646	nt5c2b	5'-nucleotidase, cytosolic IIb	389.2	2.50E-06
38	Dr.560	fbln1	Fibulin 1	387.4	4.79E-05
39	Dr.15501	cyr61	cystein-rich, angiogenic inducer, 61	365.6	2.04E-03
40	Dr.75720	myl6	Myosin, light chain 6	356.6	1.38E-03
41	Dr.80990	htra1b	HtrA serine peptidase 1b	353.3	3.41E-04
42	Dr.76054	tpm4	Tropomyosin 4	350.0	1.82E-03
43	Dr.83404	csrp1a	Cysteine and glycine-rich protein 1a	349.3	3.08E-06
44	Dr.118039	tnmd	tenomodulin	341.5	3.31E-06
45	Dr.75641	cnn2	Calponin 2	339.9	3.35E-03
46	Dr.104797	cdc42	Cell division cycle 42	319.7	1.00E-02
47	Dr.26461	gpc1	Glypican 1	309.2	2.72E-03
48	Dr.76307	cav1	Caveolin 1	292.7	1.51E-02
49	Dr.94036	pthlh	Parathyroid hormone-like hormone	270.1	1.15E-04
50	Dr.11532	fbxo32	F-box protein 32	264.6	1.10E-02

*Sparc* encodes a prototypic matricellular protein, which is conserved in a wide variety of evolutionarily diverse organisms [20], [21]. Sparc can bind calcium, hydroxyapatite, and multiple types of collagens [22]. In mammals, Sparc is highly expressed in many developing tissues, including heart, thymus, lung, and gut [23], [24]. However, upon organ maturation, levels of Sparc decrease and remain relatively low in most adult tissues with the exception of those undergoing high rates of matrix production and proliferation such as bone, skin and gut epithelia. Moreover, there is robust elevation of Sparc expression upon injury, particularly those associated with excessive deposition of collagen [25]. Hence, expression patterns of Sparc are consistent with a critical role of this protein in collagen production and deposition, as collagen is also highly expressed in the swimbladder.

*Dcn* and *chad* belong to another glycoprotein family, the small leucine-rich repeat proteoglycan (SLRP) family. The SLRP family is found in a variety of extracellular matrix tissues, including bone, cartilage and tendon. Dcn is known to bind to different types of collagens [26]. It can be located in the ECM or in the cell membrane interacting with cell surface receptors. In muscles, Dcn located in the ECM function as components of it, regulating the matrix structure as well as modulating the bioavailability of several growth factors, including BMP-4 and TGF-b [27], [28], [29]. Overexpression of Dcn can induce migration of fibroblasts. A number of the intracellular regulators and effectors involved in cell migration can be up-regulated, including the focal adhesion proteins, and some of the small Rho GTPase such as RhoA, Rac1 and Cdc42 [30].

The second most abundant category in the top 50 transcribed genes list was cytoskeleton genes, especially those important for muscle contraction. The third most abundant category was membrane protein genes, including immune-related genes. Bacterial and fungal infections of the swimbladder are occasionally reported in various fish species [31], [32]. Having an open swimbladder that connects to the gastro-intestinal tract, the zebrafish swimbladder is more vulnerable to infection than physoclistous fishes. Our observation indicated that the swimbladder had its own defensive mechanism by expressing high levels of surface recognition molecules.

### Top enriched transcription factors in the swimbladder

Next, we compiled the list of top transcribed transcription factors in the swimbladder enriched gene list based on Gene Ontology (Table 2).

**Table 2:** Top 20 enriched transcription factors in the swimbladder.

	UGCluster	Gene Symbol	Gene Name	RPKM	P value*
1	Dr.132201	hoxc8a	Homeo box C8a	185.8	8.06E-05
2	Dr.132203	hoxc6a	Homeo box C6a	143.2	NA
3	Dr.15390	foxl1	Forkhead box L1	108.0	1.45E-03
4	Dr.89399	foxf1	Forkhead box F1	85.8	4.48E-04
5	Dr.12437	tsc22d3	TSC22 domain family, member 3	74.2	4.84E-03
6	Dr.155563	atf1	Activating transcription factor 1	63.0	4.85E-04
7	Dr.81025	foxk1	Forkhead box K1	61.5	2.41E-02
8	Dr.152531	foxq1l	Forkhead box Q, like	46.6	NA
9	Dr.139	tgif1	TGFB-induced factor homeobox 1	45.9	1.96E-02
10	Dr.80310	stat6	Signal transducer and activator of transcription 6, interleukin-4 induced	44.6	1.33E-02
11	Dr.82149	zhx3	Zinc fingers and homeoboxes 3	33.3	2.45E-02
12	Dr.20916	isl2b	Islet2b	28.0	NA
13	Dr.510	hoxc4a	Homeo box C4a	27.5	2.47E-03
14	Dr.15663	cebpg	CCAAT/enhancer binding protein (C/EBP), gamma	26.8	1.28E-02
15	Dr.32618	hoxa3a	Homeo box A3a	26.1	1.71E-03
16	Dr.91917	mnx1	Motor neuron and pancreas homeobox 1	25.9	1.63E-03
17	Dr.80606	creb3l2	cAMP responsive element binding protein 3-like 2	22.4	3.49E-03
18	Dr.8233	tbx2b	T-box 2b	22.0	1.03E-02
19	Dr.83529	hsf5	heat shock transcription factor family member 5	20.6	1.46E-03
20	Dr.114796	foxm1l	Forkhead box M1-like	17.0	7.81E-03

*NA, P value not available because the transcripts were detected only in the swimbladder.ummary of sequencing results.f Gene Ontology terms in the swimbladder enriched gene list.

**Figure 5:**
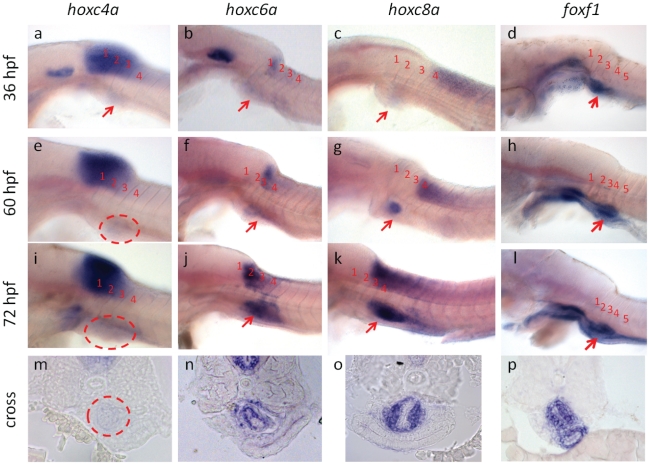
Expression of *hoxc4a*, *hoxc6a* and *hoxc8a* in developing zebrafish swimbladder. (a, e, i, m). Expression of *hoxc4a* in the swimbladder at 36 hpf (**a**), 60 hpf (**e**) and 72 hpf (**i, m**). (**b, f, j, n**) Expression of *hoxc6a* in the swimbladder at 36 hpf (**b**), 60 hpf (**f**) and 72 hpf (**j, n**). (**c, g, k, o**) Expression of *hoxc8a* in the swimbladder at 36 hpf (**c**), 60 hpf (**g**) and 72 hpf (**k, o**). (**d, h, l, p**) Expression of foxf1 in the swimbladder at 36 hpf (**d**), 60 hpf (**h**), and 72 hpf (**l, p**). Panels (**a–l**) are lateral view of embryos after whole mount in situ hybridization and panels (**m–p**) are cross-sections of in situ hybridized embryos. Swimbladder is indicated by red dashed-line circles or red arrows. Numbers are used to mark the position of somite 1–4.

One unique observation is that three genes from the hoxC cluster are enriched in the swimbladder, including *hoxc8a*, *hoxc6a* and *hoxc4a*. In order to confirm the expression of hoxC genes in swimbladder, we examined the expression of *hoxc4a*/*6a*/*8a* during zebrafish embryogenesis (Figure 5). The early expression pattern was consistent with previously reported results [33]. Expression of these genes in the notochord all had clear anterior boundaries, following the colinearity rule. *Hoxc4a* started to express in the notochord at the position of hindbrain, while *hoxc6a* and *hoxc8a* have the anterior expression boundary at approximately somite 2 and 4 respectively. None of their expression domain had a clear posterior boundary. Expression of all three genes in the swimbladder primordium could be detected at 36 hpf. The expression of *hoxc8a* became very prominent in the swimbladder starting from 48 hpf and was persistent at least until 72 hpf. Cross-section confirmed that *hoxc8a* was expressed strongly in the mesenchyme and relatively weakly in the mesothelium. *Hoxc6a* was expressed at a slightly lower level from 48 hpf to 72 hpf, and it was also expressed in the swimbladder mesenchyme and mesothelium. *Hoxc4a* was expressed at a barely visible level in the swimbladder and likely also in the mesoderm, though the exact expression domain could not be confirmed by cross-section because of its weak expression.

Two closely related Forkhead homeobox genes, *foxl1* and *foxf1* were also on the top of the list of enriched transcription factors [34]. *Foxf1* was expressed in the swimbladder primordium as early as 36 hpf (Figure 5d) and the expression was persistent in the swimbladder until at least 72 hpf (Figure 5i). At the same time, prominent expression was also observed along the alimentary tract. Cross-section confirmed that the expression of *foxf1* was restricted to the mesenchyme layer in both the swimbladder and the alimentary tract (Figure 5p). However, although the expression level of *foxl1* is higher than *foxf1* in the adult swimbladder as revealed by the RNA-seq data, expression of *foxl1* was not detected in the developing swimbladder by in situ hybridization (data not shown).

### Resemblance of swimbladder transcriptome to mammalian lung

**Figure 6:**
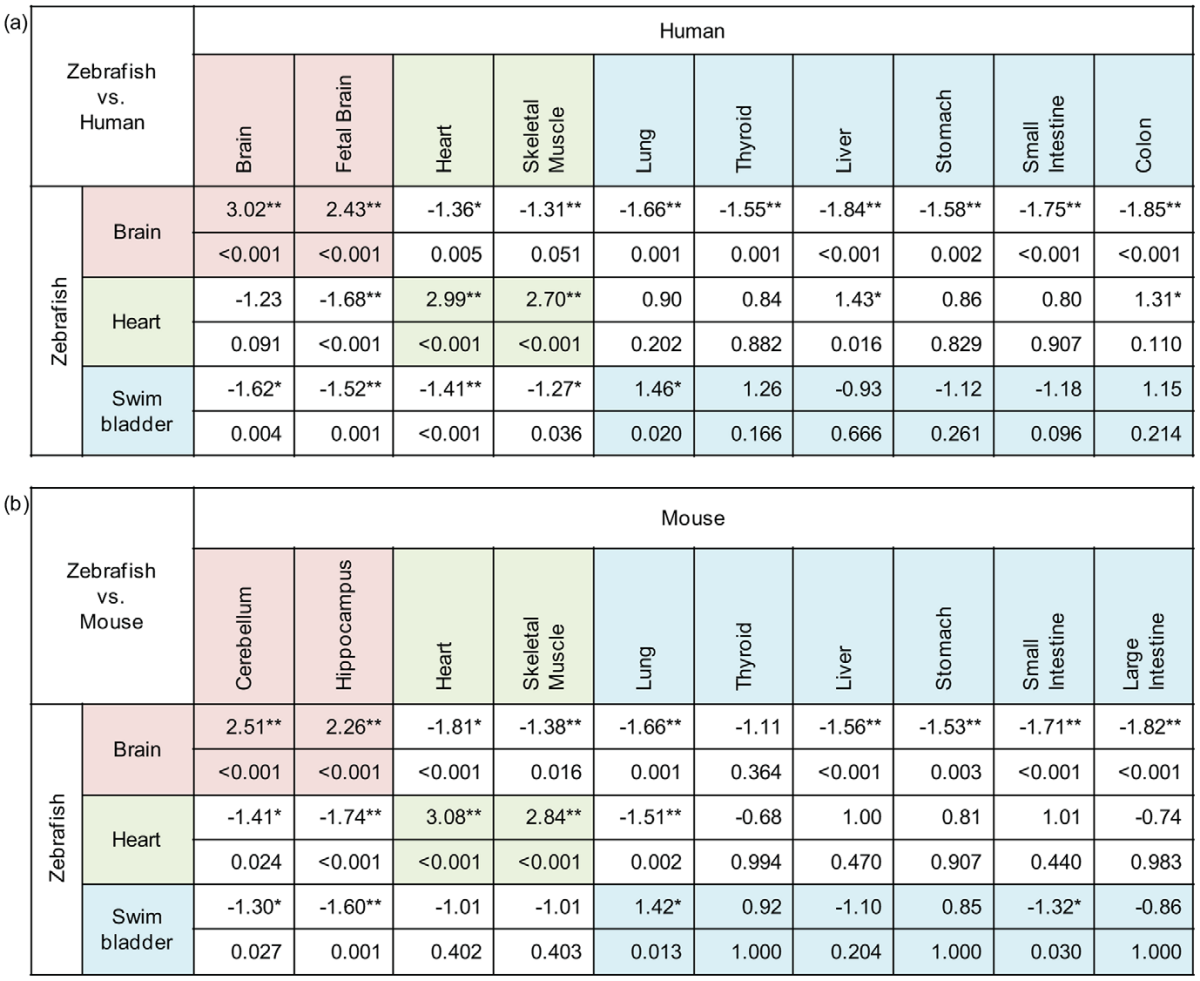
Comparison of zebrafish and human (a) or mouse (b) transcriptome tissues by GSEA. Each intersection of the two zebrafish and human or mouse tissues was split into two cells. Upper cell shows NES, and lower cell shows the corresponding FDR. **: very significant (p<0.001), *: significant (p<0.05).

In order to gain insight into molecular resemblance of fish swimbladder and mammalian lung, our swimbladder transcriptomic data were compared with the transcriptomic data from various human and mouse tissues based on microarray studies. The enriched gene list of each zebrafish tissue was used to represent its transcriptome. As shown in Figure 6, based on normalized enrichment scores (NES), the zebrafish brain show high resemblance to the human fetal and adult brains as well as the cerebellum and hippocampus of mouse. Meanwhile, the zebrafish heart closely resembles the mammalian heart and skeletal muscle, indicating similar cellular constitutions of the two tissues and thus validating the methodology. Among all the endodermal organs compared, it is interesting to note from Figure 6 that the zebrafish swimbladder has the highest and significant NES to both human and mouse lung, indicating that indeed the fish swimbladder has the highest resemblance with lung at the transcriptome level.

To further analyze the molecular resemblance of swimbladder and lung, GSEA leading edge genes, i.e., zebrafish swimbladder enriched genes appearing in the ranked list of human lung transcriptome at or before the point at which the running sum score reaches its maximum deviation from zero [35], were examined and presented in Table S7. These leading edge genes contains both constitution of the ECM (LUM, FN1, COL1A2, CYR61 and SPARC) and regulators of the ECM (TFPI, MMP2, RNPEP and HPSE), indicating that the zebrafish swimbladder and human lung may have some similar ECM characteristics. A few molecules belong to the small GTPase signaling pathway are identified (TNFAIP1, RND3, ARHGAP29, RASL12 and MX1), suggesting that the small GTPase signaling pathway may play an important role in both organs. Besides, genes involved in MAPK (DUSP1), TGF (TGFBI), and BMP (BMP5) signaling pathways are also identified. Several transcription factors are present in the list, including TGIF1, FOXF2, FOXF1, AATF and PFDN1. Examination of the leading edge gene list between zebrafish swimbladder and mouse lung showed a similar profile (data not shown).

## Discussion

### Epithelial tight junctions allow selective permeability of the swimbladder

The epithelium is the inner most layer of the swimbladder and is in direct contact with the gas inside. It has been shown by transmitted electron microscopy that the swimbladder epithelial cells are polarized even prior to inflation [36]. Tight junctions serve to form seals between epithelial cells, creating a selectively permeable barrier to intercellular diffusion. Consistent with this, our KEGG pathway analysis indicated that the tight junction pathway genes were indeed enriched in the swimbladder. Among the swimbladder enriched gene, the zebrafish homologs of *cldn4*/*5*/*6*/*7*/*9* were identified, together with members of the Rho small GTPase subfamily including *cdc42*, *rhoA* and *rab13*. Claudins are transmembrane proteins which act in concert with other transmembrane and peripheral proteins to form the physical basis for tight junction. There are roughly two dozens of different claudins. In human airways, both bronchi and bronchioles express Claudin 1, 3, 4, 5 and 7. Particularly, *CLDN3/4/5* have been found to be co-expressed by type II alveolar epithelial cells [37]. It has been revealed by immunofluorescence staining that CLDN4 is increasingly localized to the apical tight junction region, but with lower expression at the lateral region [38]. In contrast, CLDN3 and 5 are localized exclusively in the apical-most region of the tight junctions. Altered Claudin expression pattern can change the paracellular permeability characteristics of the epithelium. For example, CLDN3 overexpression decreases solute permeability, whereas CLDN5 increases permeability [39]. In summary, the expression of *CLDN*/*cldn 4*, *5* and *7* is conserved between the human lung and the zebrafish swimbladder.

However, *cldn9*, which is the one of the highest expressed in the swimbladder, is not identified in the human lung. Interestingly, *Cldn9* is the most highly expressed in the inner ear of all the Claudin family members [40], and it is present in all of the major epithelial cell types that line the endolymphatic space. Analysis of *Cldn9* mutant mice shows that Cldn9 is a paracellular ion permeability barrier for Na^+^ and K^+^, and loss of *Cldn9* expression in the inner ear disrupts the Na^+^/K^+^ barrier and causes deafness. In contrast, a mutant zebrafish line with K^+^ channel defect shows both hearing defect and swimbladder over-inflation [10], suggesting that K^+^ channel plays a very important role in regulating swimbladder volume. In the zebrafish, the larvae surface and swallow a bolus of air, which is passed down through the esophagus and into the swimbladder via the pneumatic duct, to inflate their swimbladders [41]. However, how the larvae and adult fish maintain and regulate the swimbladder volume is unclear and seems to be independent of surface contact. Based on these findings, we speculate that *cldn9* is likely to be involved in forming a Na^+^/K^+^ barrier in the swimbladder and to regulate swimbladder volume. It is also interesting to note that swimbladder has long been recognized to function for sound production and hearing [42].

### Smooth muscle regulation and the ECM

It has been previously revealed by phalloidin labeling of muscle fibers revealed that smooth muscles are the major muscle constitution in the swimbladder and myocytes form thick bands along the ventral surface of the anterior chamber and bilaterally along the posterior chamber. In contrast, striated muscle fibers constitute a sphincter at the junction of the esophagus with the pneumatic duct [43]. The abundance of muscle-related genes identified in the swimbladder transcriptome correlates with this feature. Besides, KEGG pathway and GSEA analysis showed critical role of interaction between the cells and surrounding extracellular matrix.

The viscoelasticity of smooth muscle is contributed by a complex extracellular matrix. The ECM is not only a supporting structure of the smooth muscles, but also a dynamic structure constantly turning over its contents. This explains the abundant ECM-relating transcripts and the active protein transportation process. The major protein constituting ECM are collagens, glycoproteins and proteoglycans. In our transcriptome data, we also observed these transcripts expressing at high levels in the swimbladder. Collagen I is the only type of collagen identified in the swimbladder transcriptome, and it is also the most abundant collagen in the human body. In mammalian tissues, type I collagen shows the highest expression in the cardiomyocytes and smooth muscles [44].

Previously, it has been reported that human airway smooth muscle cells in culture can secrete various ECM proteins [45], [46]. The ECM can store inflammatory mediators and growth factors, which can be released via the action of MMPs (matrix metalloproteinases) to modulate smooth muscle proliferative and synthetic capacity. The composition of the ECM can be regulated by the synthesis of new proteins, and by the action of MMPs and TIMPs (tissue inhibitor of metalloproteinases). In the swimbladder, *mmp2* and *timp2* are the only MMP and TIMP identified. Mmp2 functions to degrade type IV collagen, which is a major structural component of the basement membranes. The activity of Mmp2 is often associated with excessive extracellular turnover, which is consistent with our observations that *sparc* is the most abundant transcript in the swimbladder. Interestingly, TIMP2 has been shown to be able to directly bind and inhibit MMP2 activity [47]. Therefore, *mmp2* and *timp2* may function to balance the extracellular turnover rate in the swimbladder.

### Possible roles of *hoxC* family genes in the swimbladder

Hox genes are one of the master regulators of pattern formation during embryogenesis. They regulate pattern formation by coordinating cell proliferation, migration, adhesion and differentiation. Our data on the embryonic expression pattern of hoxC family members and the adult transcriptome data together suggest that the expression of embryonic hox genes is persistent until adult stage. This is in consistent with the previous findings that hox genes might have an enduring role in maintaining positional identity throughout the lifetime of an organism [48], [49]. As the expression of *hoxc4a*/*6a*/*8a* in developing swimbladder was identified, the function of these genes remains an open question; thus, it is worth further exploring their regulatory mechanisms in future studies.

In humans, *HOXC6* mRNA is detected in both fetal and normal adult lung. On contrary, *HOXC8* mRNA is present in the fetal lung, but absent from normal adult lung. Interestingly, *HOXC8* is consistently up-regulated in emphysematous lungs, a disease in which the alveolar septum is disintegrated and the alveoli gradually lose the elasticity. However, the human lung has a different expression profile of Hox genes. In both human fetal and adult lungs, the most abundant expressed Hox genes are *HOXA5, HOXB2* and *HOXB5*. Among these genes, only the homolog of *HOXB5* is expressed in the zebrafish swimbladder at a relatively low level. It is mostly accepted that the swimbladder and lung were evolved from the same ancestral organ, namely the respiratory pharynx. The swimbladder arises from the dorsal part, while the lung originated from the ventral part. The different expression profiles of *HOX/hox* genes in the swimbladder and lung are consistent with this double origin theory.

In recent years, it becomes increasingly clear that hox genes have regulatory roles in the adult, likely involved in cell renewal and in the normal physiological changes that occur in the adult life [50]. The deregulated expression of hox genes in adulthood is associated with cancer development and malignant progression such as invasion and metastasis [51], [52]. Noticeably, *HOXC* cluster genes have been shown to be selectively overexpressed in prostate carcinoma and may play key roles in the acquisition of invasive and metastatic phenotypes of prostate cancer cells [53], [54]. Both of *Hoxc6* and *Hoxc8* have been shown to be able to regulate the cross-talk between Wnt, BMP, and FGF signaling pathways by directly targeting a few important regulators in the pathways [55], [56], [57]. Thus, the expression of *HoxC* cluster genes in the swimbladder may not only serve to memorize the positional identity of epithelial cells, but also act as master regulator for adult swimbladder function, likely in cellular adhesion and mobility.

### Evolutionary insights between the fish swimbladder and mammalian lung

Epithelial cells of air-breathing organs of vertebrates are covered with a thin layer surfactant, which reduces and modifies surface tension at the air-liquid interphase. Surfactant consists of mixtures of lipids and surfactant proteins (SPs). In humans, four surfactant proteins have been identified: SP-A, SP-B, SP-C, and SP-D. These four proteins belong to three different superfamilies. Both SP-A and SP-D are collectins, and they are known to play a role in innate immune defense of the lungs by binding a wide array of pathogens, including viruses, bacteria, and fungi, and facilitating their uptake by immune cells. Both of them are rooted by the MBL (Mannose binding lectin) sequence [58]. Homologs of SP-A has been identified in the swimbladder of goldfish by western and northern blot analyses [59]. We also identified a zebrafish homolog in this family, *lman2 (lectin,manose binding2)*, expressed in the swimbladder, which was confirmed by real-time qRT-PCR (Figure 2). SP-B, which is highly hydrophobic, belongs to the superfamily of saposin-like proteins, a diverse group of lipid-interacting proteins. We identified prosaposin (Dr.75922) transcripts in the zebrafish swimbladder at intermediate abundance (78.9 RPKM), and it is also enriched in the swimbladder. SP-C belongs to the chondromodulin I (CHM1) family. One of the zebrafish homolog from the gene family, tenomodulin (Dr.118039, 341.47 RPKM) is highly transcribed and enriched in the swimbladder. Taken together, the homologs of all four human SPs have been identified in the zebrafish swimbladder transcriptome, further supporting the evolutionary relationship of the fish swimbladder and mammalian lung. In human lung, SP-A is the most dominant surfactant protein expressing [60]. However, in the zebrafish swimbladder transcriptome, homologs of SP-B and SP-C are highly expressed surfactant-related genes. Since both of them are hydrophobic, the higher expressing level may due to the fact that the fraction of lipid (mainly cholesterol) in the swimbladder is higher than in lung surfactant of mammals [61]. In contrast to lung surfactant, swimbladder surfactant mainly acts as an antiglue to facilitate reopening of the swimbladder after a collapse or partial collapse, and it may prevent edema [62].

Gas gland cells of physostome have been shown to produce surfactant in vivo and in culture [63]. Lamellar bodies are also observed in the apical region of these cells. No anatomical evidence for a gas gland was found in the zebrafish swimbladder in previous study [43]. However, many species of physostomes that are known to secrete gas into their swimbladders do not have a morphologically identifiable gas gland, and it has been proposed that the gas-secreting cells may be scattered singly or in small groups in the wall of the swimbladder in these species [64], [65].Immunohistochemistry staining suggested the presence of gas-secreting cells in the zebrafish swimbladder by showing nerve terminal concentration of autonomic nerve terminals [43].

Another clue of the evolutionary homology is the parathyroid hormone-related protein (PTHrP). Ligand-receptor signaling involving PTHrP is crucial for the development and proper functioning of lungs in all vertebrates studied. Its expression correlates with lung maturation, homeostasis, and repair as well as alveolar size, septal thickness and composition of the matrix [66]. It is expressed throughout vertebrate phylogeny, beginning with its expression in the fish swimbladder as an adaption to gravity. The zebrafish swimbladder transcriptome provides supporting evidence by showing the high expression of parathyroid hormone (*pth*, Dr.94036).

## Materials and Methods

### Ethics statement

All experimental protocols were approved by Institutional Animal Care and Use Committee (IACUC) of National University of Singapore (Protocol 079/07).

### RNA sample preparation and library sequencing

Healthy Singapore wildtype adult zebrafish (around 6 months old) were purchased from a local fish farm. The swimbladders including the attached pneumatic ducts were isolated from 45 female and 45 male fish and pooled. Brains, hearts and head kidneys were also collected from the same batch of fish for comparative studies. Total RNA was extracted using TRIzol® Reagent (Invitrogen). mRNA (polyA+) was purified using DynaBeads® Oligo(dT)25 (Invitrogen) according to the manufacture’s protocol and treated with DNaseI (Ambion)to remove DNA contamination. The resulted mRNA sample was quantified on NanoDrop® ND-100 Spectrophotometer (Thermo Scientific). Prior to cDNA synthesis, mRNAs were hydrolyzed by RNA Fragmentation Reagent (Ambion). Paired-ends sequencing was performed using Sanger-modified Illumina protocol [67], [68].

We used MAQ (Mapping and Assembly with Qualities) to align the sequence tags to transcriptome database [69]. MAQ assign each alignment a *phred*-scaled quality score (Qs), which measures the probability that the true alignment is not the one found by MAQ. The data have been submitted to the European Bioinformatics Institute (EBI) database (Accession number: ERP000447). ZGC database (retrieved on Jan 28, 2011) was used in this study, which contains 16,739 ORFs (Open Reading Frames). The sequencing results were summarized in Table S1. The mapped sequence tags for each transcript entry were normalized into RPKM as previously described [18].

### Annotation

To facilitate functional implications of zebrafish transcriptome, all zebrafish genes were mapped to annotated human and mouse genes in order to use existing online software developed in human genes. Thus, Unigene annotation of zebrafish transcript entries (GenBank accession ID) and human and mouse homology mapping of zebrafish Unigene clusters were retrieved from the Genome Institute of Singapore Zebrafish Annotation Database (http://giscompute.gis.a-star.edu.sg/~govind/unigene_db/) as previously described [70]. For Unigene clusters mapped by more than one transcript entries, the highest RPKM was used to represent the expression level of the Unigene cluster [71]. In this study, the transcript entries of the ZGC database were mapped to 6392 unique human Unigene clusters and 6793 unique mouse Unigene clusters. Some zebrafish Unigene clusters were mapped to more than one human or mouse Unigene clusters, which usually came from the same gene family. To remove redundancy and avoid causing bias in functional analyses, only the first human or mouse Unigene cluster in the list was selected to represent the zebrafish Unigene clusters. Functional characterization of human and mouse Unigenes clusters was based on Gene Ontology and can be obtained from Stanford’s SOURCE database [72].

### Swimbladder-enriched gene selection by t-test

While Gene Ontology analysis can provide a general picture of the swimbladder transcriptome, the unique features of the swimbladder may only be unmasked by removing those housekeeping genes which are commonly expressed in all tissues. Therefore, one-sample t-test was conducted to select enriched Unigenes in the swimbladder against other zebrafish tissues. One sample t-test was performed according to the standard method implemented in MATLAB. The *p* value is the probability, under the null hypothesis, of observing a value as extreme or more extreme of the test statistic
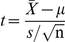
where is the sample mean or RPKM values of a transcript in the swimbladder, *µ* is the population mean or mean RKPM values of the same transcript in the other three comparing tissues, *s* is the sample standard deviation calculated from population means in the three comparing tissues, and *n* is the sample size and the value is 3 here. Unigene clusters with *p* value smaller than 0.025 are defined as enriched genes. At the same time, a second threshold of RPKM>10 and RPKM>average RPKM of the four comparing zebrafish tissues (swimbladder, brain, heart and head kidney) is added to ensure that the selected genes are relatively abundant and physiologically relevant. The enriched gene lists contain 888, 1,732 and 535 zebrafish Unigene clusters for the swimbladder, brain and heart, respectively. The lists were subsequently converted into 491, 967 and 323 homologous human Unigene clusters and 483, 963 and 311homologous mouse Unigene clusters.

### Gene Ontology slim classification and enrichment analysis

Gene ontology slim classification was performed using WebGestalt against the total ZGC database (containing 9,631 zebrafish Unigene clusters) and the total zebrafish swimbladder transcriptome (containing 5,506 zebrafish Unigene clusters). The significance level of enrichment was indicated by false discovery rate (FDR)-corrected p-value from hypergeometric test. The cutoff is FDR<0.01.

Gene ontology enrichment analysis was performed using DAVID (The Database for Annotation, Visualization and Integrated Discovery) with the total zebrafish genome information as the background and p-values representing a modified Fisher’s exact t-test. Gene Ontology Fat categories were used for this analysis. GO Fat is a term that the DAVID team used to describe a subset of the GO term set. It is coined after GO slim which serves as a subset of the broadest GO terms. In contrast, the GO Fat attempts to filter out the broadest terms so that they will not overshadow the more specific terms. FDR score was also provided as a multiple testing correction method. Unless specifically indicated, the cut-off of p-value is <0.01. KEGG pathway analysis was also performed similarly using DAVID.

### Analysis of the tissue-specific enriched gene list using GSEA pre-ranked analysis

GSEA Pre-ranked option was used to analyze the entire swimbladder enriched gene list. Briefly, the gene symbols of human homologs of the enriched zebrafish Unigene clusters were ranked using logarithm transformed p-value (base 10). The number of permutation used was 1000. Pathways with nominal p-value (NP) <0.05 were considered statistically significant.

### Cross-species and cross-platforms analysis

Two sets of transcriptome data for healthy human and mouse tissues (GSE2361 and GSE97) were obtained from GEO (Gene Expression Omnibus). Annotation information was retrieved from the Genome Institute of Singapore Annotation Database (http://giscompute.gis.a-star.edu.sg/~govind/unigene_db/). For multiple probes which can be mapped to one Unigene cluster, the maximum signal intensity was selected to represent the expression level of the Unigene cluster.

We used GSEA to establish the relatedness between zebrafish and mammalian tissues. GSEA is a computational method that determines whether a *priori* defined set of genes shows statistically significant, concordant differences between two biological samples; it calculates an enrichment score using a running-sum statistic through a ranked list of gene expression data set [35]. The zebrafish swimbladder, brain and heart transcriptome lists were converted into human and mouse homolog Unigene clusters. The enriched gene list of each tissue was used to represent its transcriptome. The statistical significance of the enrichment score was estimated by using an empirical phenotype-based permutation test procedure. An FDR value was provided by introducing adjustment of multiple hypothesis testing.

### Real-time PCR

Real-time PCR was performed using the LightCycler system (Roche Applied Science) with LightCycler FastStart DNA Master SYBR Green I (Roche Applied Science) according to the manufacturer’s instructions. cDNA was synthesized from the same RNA sample which were used for the RNA-seq. For comparison between real-time PCR and RNA-seq results, Cp and RPKM values for each gene were normalized against Cp and RPKM of *ef1a* (Dr.31797).

### Whole mount in situ hybridization

In situ hybridization probes were generated from available sequences in the public databases. The plasmids were linearized to synthesize both sense and antisense probes with T7 or SP6 RNA polymerase by using digoxigenin (DIG) RNA labeling mix (Roche Applied Science). Whole mount in situ hybridization (WISH) was performed using standard protocols as described previously [76].

## Supporting Information

Table S1 **Summary of sequencing results for the zebrafish swimbladder, brain, heart and head kidney.**
(DOC)

Table S2 **Detailed results of Gene Ontology slim classification of the entire swimbladder transcriptome.**
(DOC)

Table S3 **Complete list of zebrafish genes expressed in the swimbladder.** The genes are annotated based on Unigene cluster ID and ranked by RPKM. Swimbladder enriched genes are indicated in bold.
(XLS)

Table S4 **Enrichment of Gene Ontology terms in the swimbladder enriched gene list.** The counts are presented in Unigene cluster counts. The percentage for each GO term represents the percentage of Unigene clusters in the GO term in the total transcript entries identified in the DAVID database. P-values represent a modified Fisher’s exact t-test. Only GO terms with p-value<0.01 were shown in the table.
(DOC)

Table S5 **KEGG pathway analysis of the swimbladder enriched gene list.** The counts are presented in Unigene cluster counts. The percentage for each GO term represents the percentage of Unigene clusters in the GO term in the total transcript entries identified in the DAVID database. P-values represent a modified Fisher’s exact t-test. Only GO terms with p-value<0.05 were shown in the table.
(DOC)

Table S6 **GSEA analysis of the swimbladder enriched gene list.** Gene sets that are statistically enriched with nominal p-value (NP) are shown. The sizes of the gene sets mean the number of the genes from the pre-defined canonical pathway database which are identified from the swimbladder enriched gene list. Values of normalized enrichment score (NES) indicate the activities of the enriched gene sets.
(DOC)

Table S7 **GSEA leading edge genes between zebrafish swimbladder and human lung.**
(DOCX)
