# Supplementary material for: Comparative Transcriptome Analyses Indicate Molecular Homology of Zebrafish Swimbladder and Mammalian Lung
Source: PLoS One. 2011 Aug 26;6(8):e24019. doi: 10.1371/journal.pone.0024019 (PMC3162596; doi:10.1371/journal.pone.0024019)
Supplement: Table S1 — Summary of sequencing results for the zebrafish swimbladder, brain, heart and head kidney. (DOC) [file pone.0024019.s001.doc]

**Table S1. Summary of sequencing results for the zebrafish swimbladder, brain, heart and head kidney**

| Tissue | Total tag reads | Total transcript entries mapped | Transcript entries above 10 RPKM cutoff | Zebrafish Unigene clusters mapped above 10 RPKM cutoff |
| --- | --- | --- | --- | --- |
| Swimbladder | 34,026,474 | 9,315 | 5,758 | 5,506 |
| Brain | 38,938,626 | 8,825 | 5,587 | 5,354 |
| Heart | 42,820,495 | 7,889 | 4,180 | 4,008 |
| Head kidney | 37,039,047 | 8,910 | 4,863 | 4,662 |
