# Supplementary material for: Comparative Transcriptome Analyses Indicate Molecular Homology of Zebrafish Swimbladder and Mammalian Lung
Source: PLoS One. 2011 Aug 26;6(8):e24019. doi: 10.1371/journal.pone.0024019 (PMC3162596; doi:10.1371/journal.pone.0024019)
Supplement: Table S2 — Detailed results of Gene Ontology slim classification of the entire swimbladder transcriptome. (DOC) [file pone.0024019.s002.doc]

**Table S2. Detailed results of Gene Ontology slim classification of the entire swimbladder transcriptome**

|  | Categories | Swimbladder Unigene entries | Swimbladder Unigene percentage | Total ZGC Unigene entries | ZGC Unigene percentage | P_value | FDR |
| --- | --- | --- | --- | --- | --- | --- | --- |
| Biological Process | Metabolic process | 1477 | 32.90% | 3179 | 33.36% | 1.17E-02 | 1.84E-02 |
|  | Biological regulation | 782 | 17.42% | 1908 | 20.02% | 3.08E-10 | 1.13E-09 |
|  | Multicellular organismal process | 365 | 8.13% | 1010 | 10.60% | 2.65E-14 | 2.92E-13 |
|  | Developmental process | 349 | 7.77% | 943 | 9.90% | 1.08E-11 | 5.94E-11 |
|  | Cell communication | 370 | 8.24% | 921 | 9.66% | 1.41E-06 | 3.10E-06 |
|  | Localization | 387 | 8.62% | 921 | 9.66% | 1.38E-04 | 2.53E-04 |
|  | Response to stimulus | 189 | 4.21% | 423 | 4.44% | 2.36E-02 | 2.60E-02 |
|  | Cellular component organization | 203 | 4.52% | 408 | 4.28% | 2.21E-02 | 2.60E-02 |
|  | Death | 62 | 1.38% | 111 | 1.16% | 1.37E-02 | 1.88E-02 |
|  | Growth | 42 | 0.94% | 88 | 0.92% | 8.47E-02 | 8.47E-02 |
|  | Unclassified | 2190 | 48.79% | 4396 | 46.13% | 9.48E-08 | 2.61E-07 |
| Molecular Function | Ion binding | 661 | 14.72% | 1470 | 15.42% | 4.60E-03 | 7.23E-03 |
|  | Nucleic acid binding | 644 | 14.35% | 1398 | 14.67% | 1.62E-02 | 2.23E-02 |
|  | Protein binding | 587 | 13.08% | 1256 | 13.18% | 2.33E-02 | 2.53E-02 |
|  | Nucleotide binding | 551 | 12.27% | 1046 | 10.98% | 1.75E-05 | 3.85E-05 |
|  | Hydrolase activity | 422 | 9.40% | 915 | 9.60% | 2.28E-02 | 2.53E-02 |
|  | Transferase activity | 389 | 8.67% | 841 | 8.82% | 2.53E-02 | 2.53E-02 |
|  | Transcription regulator activity | 191 | 4.25% | 634 | 6.65% | 0.00E+00 | 0.00E+00 |
|  | Molecular transducer activity | 187 | 4.17% | 592 | 6.21% | 0.00E+00 | 0.00E+00 |
|  | Transporter activity | 138 | 3.07% | 457 | 4.80% | 1.86E-14 | 6.82E-14 |
|  | Structural molecule activity | 130 | 2.90% | 218 | 2.29% | 4.91E-05 | 9.00E-05 |
|  | Unclassified | 1889 | 42.08% | 3680 | 38.61% | 7.79E-12 | 2.14E-11 |
| Cellular Component | Membrane | 685 | 15.26% | 1790 | 18.78% | 0.00E+00 | 0.00E+00 |
|  | Nucleus | 562 | 12.52% | 1267 | 13.29% | 2.63E-03 | 4.82E-03 |
|  | Macromolecular complex | 425 | 9.47% | 772 | 8.10% | 7.28E-07 | 2.67E-06 |
|  | Ribosome | 108 | 2.41% | 201 | 2.11% | 9.36E-03 | 1.47E-02 |
|  | Mitochondrion | 104 | 2.32% | 195 | 2.05% | 1.23E-02 | 1.50E-02 |
|  | Cytoskeleton | 99 | 2.21% | 136 | 1.43% | 5.55E-10 | 3.05E-09 |
|  | Endoplasmic reticulum | 81 | 1.80% | 136 | 1.43% | 9.53E-04 | 2.10E-03 |
|  | Envelope | 74 | 1.65% | 134 | 1.41% | 1.16E-02 | 1.50E-02 |
|  | Glogi apparatus | 65 | 1.45% | 130 | 1.36% | 5.64E-02 | 5.64E-02 |
|  | Endomembrane system | 54 | 1.20% | 97 | 1.02% | 1.94E-02 | 2.13E-02 |
|  | Unclassified | 2893 | 64.45% | 5954 | 62.48% | 1.50E-05 | 4.13E-05 |
