# Supplementary material for: Comparative Transcriptome Analyses Indicate Molecular Homology of Zebrafish Swimbladder and Mammalian Lung
Source: PLoS One. 2011 Aug 26;6(8):e24019. doi: 10.1371/journal.pone.0024019 (PMC3162596; doi:10.1371/journal.pone.0024019)
Supplement: Table S4 — Enrichment of Gene Ontology terms in the swimbladder enriched gene list. The counts are presented in Unigene cluster counts. The percentage for each GO term represents the percentage of Unigene clusters in the GO term in the total transcript entries identified in the DAVID database. P-values represent a modified Fisher’s exact t-test. Only GO terms with p-value<0.01 were shown in the table. (DOC) [file pone.0024019.s004.doc]

**Table S4.** Enrichment of Gene Ontology terms in the swimbladder enriched gene list

|  | Term | Count | % | PValue |
| --- | --- | --- | --- | --- |
| Biological Process | GO:0001558~regulation of cell growth | 12 | 1.20 | 1.56E-05 |
|  | GO:0040008~regulation of growth | 12 | 1.20 | 6.05E-05 |
|  | GO:0048729~tissue morphogenesis | 15 | 1.50 | 3.16E-03 |
|  | GO:0007242~intracellular signaling cascade | 38 | 3.79 | 3.55E-03 |
|  | GO:0042074~cell migration involved in gastrulation | 11 | 1.10 | 4.36E-03 |
|  | GO:0016477~cell migration | 16 | 1.60 | 4.82E-03 |
|  | GO:0007264~small GTPase mediated signal transduction | 22 | 2.20 | 5.40E-03 |
|  | GO:0051674~localization of cell | 17 | 1.70 | 5.51E-03 |
|  | GO:0048870~cell motility | 17 | 1.70 | 5.51E-03 |
|  | GO:0001667~ameboidal cell migration | 13 | 1.30 | 6.30E-03 |
|  | GO:0051216~cartilage development | 9 | 0.90 | 7.67E-03 |
|  | GO:0051169~nuclear transport | 6 | 0.60 | 8.44E-03 |
|  | GO:0006913~nucleocytoplasmic transport | 6 | 0.60 | 8.44E-03 |
|  | GO:0048598~embryonic morphogenesis | 23 | 2.30 | 9.56E-03 |
|  | GO:0060026~convergent extension | 9 | 0.90 | 9.67E-03 |
| Molecular Function | GO:0005520~insulin-like growth factor binding | 10 | 1.00 | 6.74E-06 |
|  | GO:0019838~growth factor binding | 10 | 1.00 | 7.56E-05 |
|  | GO:0005525~GTP binding | 34 | 3.39 | 3.01E-03 |
|  | GO:0032561~guanyl ribonucleotide binding | 34 | 3.39 | 3.60E-03 |
|  | GO:0019001~guanyl nucleotide binding | 34 | 3.39 | 3.91E-03 |
| Cellular Component | GO:0005576~extracellular region | 54 | 5.39 | 4.58E-08 |
|  | GO:0005578~proteinaceous extracellular matrix | 16 | 1.60 | 1.20E-04 |
|  | GO:0031012~extracellular matrix | 16 | 1.60 | 1.98E-04 |
|  | GO:0044421~extracellular region part | 21 | 2.10 | 1.12E-03 |
|  | GO:0043235~receptor complex | 6 | 0.60 | 5.00E-03 |
|  | GO:0005783~endoplasmic reticulum | 24 | 2.40 | 7.78E-03 |

The counts are presented in Unigene cluster counts. The percentage for each GO term represents the percentage of Unigene clusters in the GO term in the total transcript entries identified in the DAVID database. P-values represent a modified Fisher’s exact t-test. Only GO terms with p-value<0.01 were shown in the table.
