# Supplementary material for: Comparative Transcriptome Analyses Indicate Molecular Homology of Zebrafish Swimbladder and Mammalian Lung
Source: PLoS One. 2011 Aug 26;6(8):e24019. doi: 10.1371/journal.pone.0024019 (PMC3162596; doi:10.1371/journal.pone.0024019)
Supplement: Table S5 — KEGG pathway analysis of the swimbladder enriched gene list. The counts are presented in Unigene cluster counts. The percentage for each GO term represents the percentage of Unigene clusters in the GO term in the total transcript entries identified in the DAVID database. P-values represent a modified Fisher’s exact t-test. Only GO terms with p-value<0.05 were shown in the table. (DOC) [file pone.0024019.s005.doc]

**Table S5. KEGG pathway analysis of the swimbladder enriched gene list**

| Term | Count | % | PValue |
| --- | --- | --- | --- |
| dre04510:Focal adhesion | 29 | 2.89 | 3.22E-06 |
| dre04810:Regulation of actin cytoskeleton | 24 | 2.40 | 9.11E-04 |
| dre04512:ECM-receptor interaction | 11 | 1.10 | 1.73E-03 |
| dre04520:Adherens junction | 12 | 1.20 | 8.45E-03 |
| dre04530:Tight junction | 14 | 1.40 | 2.74E-02 |
| dre04340:Hedgehog signaling pathway | 8 | 0.80 | 3.54E-02 |

The counts are presented in Unigene cluster counts. The percentage for each GO term represents the percentage of Unigene clusters in the GO term in the total transcript entries identified in the DAVID database. P-values represent a modified Fisher’s exact t-test. Only GO terms with p-value<0.05 were shown in the table.
