# Supplementary material for: Comparative Transcriptome Analyses Indicate Molecular Homology of Zebrafish Swimbladder and Mammalian Lung
Source: PLoS One. 2011 Aug 26;6(8):e24019. doi: 10.1371/journal.pone.0024019 (PMC3162596; doi:10.1371/journal.pone.0024019)
Supplement: Table S6 — GSEA analysis of the swimbladder enriched gene list. Gene sets that are statistically enriched with nominal p-value (NP) are shown. The sizes of the gene sets mean the number of the genes from the pre-defined canonical pathway database which are identified from the swimbladder enriched gene list. Values of normalized enrichment score (NES) indicate the activities of the enriched gene sets. (DOC) [file pone.0024019.s006.doc]

**Table S6. GSEA analysis of the swimbladder enriched gene list**

| NAME | SIZE | NES | NOM p-val |
| --- | --- | --- | --- |
| HSA04512_ECM_RECEPTOR_INTERACTION | 9 | 1.71 | 1.63E-02 |
| HSA04060_CYTOKINE_CYTOKINE_RECEPTOR_INTERACTION | 8 | 1.68 | 3.03E-02 |
| HSA04340_HEDGEHOG_SIGNALING_PATHWAY | 6 | 1.62 | 3.58E-02 |
| HSA04350_TGF_BETA_SIGNALING_PATHWAY | 10 | 1.56 | 4.64E-02 |

Gene sets that are statistically enriched with nominal p-value (NP) are shown. The sizes of the gene sets mean the number of the genes from the pre-defined canonical pathway database which are identified from the swimbladder enriched gene list. Values of normalized enrichment score (NES) indicate the activities of the enriched gene sets.
