# Supplementary material for: Comparative Transcriptome Analyses Indicate Molecular Homology of Zebrafish Swimbladder and Mammalian Lung
Source: PLoS One. 2011 Aug 26;6(8):e24019. doi: 10.1371/journal.pone.0024019 (PMC3162596; doi:10.1371/journal.pone.0024019)
Supplement: Table S7 — GSEA leading edge genes between zebrafish swimbladder and human lung. (DOCX) [file pone.0024019.s007.docx]

**Table S7. GSEA leading edge genes between zebrafish swimbladder and human lung**

|  | UGCluster | Symbol | Name |
| --- | --- | --- | --- |
| 1 | Hs.437322 | TNFAIP6 | Tumor necrosis factor, alpha-induced protein 6 |
| 2 | Hs.200738 | SLC38A6 | Solute carrier family 38, member 6 |
| 3 | Hs.494496 | FBP1 | Fructose-1,6-bisphosphatase 1 |
| 4 | Hs.74034 | CAV1 | Caveolin 1, caveolae protein, 22kDa |
| 5 | Hs.517168 | TAGLN2 | Transgelin 2 |
| 6 | Hs.200804 | SDCBP | Syndecan binding protein (syntenin) |
| 7 | Hs.406475 | LUM | Lumican |
| 8 | Hs.494173 | ANXA1 | Annexin A1 |
| 9 | Hs.76686 | GPX1 | Glutathione peroxidase 1 |
| 10 | Hs.373550 | TGIF1 | TGFB-induced factor homeobox 1 |
| 11 | Hs.516578 | TFPI | Tissue factor pathway inhibitor |
| 12 | Hs.446017 | WSB1 | WD repeat and SOCS box containing 1 |
| 13 | Hs.76090 | TNFAIP1 | Tumor necrosis factor, alpha-induced protein 1 (endothelial) |
| 14 | Hs.203717 | FN1 | Fibronectin 1 |
| 15 | Hs.235069 | RECQL | RecQ protein-like (DNA helicase Q1-like) |
| 16 | Hs.369397 | TGFBI | Transforming growth factor, beta-induced, 68kDa |
| 17 | Hs.103755 | RIPK2 | Receptor-interacting serine-threonine kinase 2 |
| 18 | Hs.6838 | RND3 | Rho family GTPase 3 |
| 19 | Hs.17518 | RSAD2 | Radical S-adenosyl methionine domain containing 2 |
| 20 | Hs.496622 | PLS3 | Plastin 3 |
| 21 | Hs.380627 | CMTM6 | CKLF-like MARVEL transmembrane domain containing 6 |
| 22 | Hs.523004 | PSAP | Prosaposin |
| 23 | Hs.145717 | JMJD5 | Jumonji domain containing 5 |
| 24 | Hs.490415 | ZYX | Zyxin |
| 25 | Hs.513617 | MMP2 | Matrix metallopeptidase 2 |
| 26 | Hs.126550 | VPS4B | Vacuolar protein sorting 4 homolog B |
| 27 | Hs.436142 | PTPN13 | Protein tyrosine phosphatase, non-receptor type 13 |
| 28 | Hs.234642 | AQP3 | Aquaporin 3 (Gill blood group) |
| 29 | Hs.497391 | RNPEP | Arginyl aminopeptidase (aminopeptidase B) |
| 30 | Hs.322901 | UTP3 | UTP3, small subunit (SSU) processome component, homolog |
| 31 | Hs.309090 | SRSF7 | Serine/arginine-rich splicing factor 7 |
| 32 | Hs.483238 | ARHGAP29 | Rho GTPase activating protein 29 |
| 33 | Hs.108029 | SH3BGRL | SH3 domain binding glutamic acid-rich protein like |
| 34 | Hs.59332 | SPRED2 | Sprouty-related, EVH1 domain containing 2 |
| 35 | Hs.484423 | FOXF2 | Forkhead box F2 |
| 36 | Hs.489142 | COL1A2 | Collagen, type I, alpha 2 |
| 37 | Hs.8867 | CYR61 | Cysteine-rich, angiogenic inducer, 61 |
| 38 | Hs.155591 | FOXF1 | Forkhead box F1 |
| 39 | Hs.171695 | DUSP1 | Dual specificity phosphatase 1 |
| 40 | Hs.529272 | MARCH7 | Membrane-associated ring finger (C3HC4) 7 |
| 41 | Hs.474010 | PTTG1IP | Pituitary tumor-transforming 1 interacting protein |
| 42 | Hs.296648 | BMP5 | Bone morphogenetic protein 5 |
| 43 | Hs.9196 | C9orf156 | Chromosome 9 open reading frame 156 |
| 44 | Hs.79110 | NCL | Nucleolin |
| 45 | Hs.406096 | ZFAND5 | Zinc finger, AN1-type domain 5 |
| 46 | Hs.195740 | AATF | Apoptosis antagonizing transcription factor |
| 47 | Hs.120766 | ZNF330 | Zinc finger protein 330 |
| 48 | Hs.436037 | MYOC | Myocilin, trabecular meshwork inducible glucocorticoid response |
| 49 | Hs.27018 | RASL12 | RAS-like, family 12 |
| 50 | Hs.527861 | OS9 | Osteosarcoma amplified 9, endoplasmic reticulum lectin |
| 51 | Hs.180909 | PRDX1 | Peroxiredoxin 1 |
| 52 | Hs.44227 | HPSE | Heparanase |
| 53 | Hs.25441 | KPTN | Kaptin (actin binding protein) |
| 54 | Hs.426312 | AMOTL2 | Angiomotin like 2 |
| 55 | Hs.515371 | CAPNS1 | Calpain, small subunit 1 |
| 56 | Hs.111779 | SPARC | Secreted protein, acidic, cysteine-rich (osteonectin) |
| 57 | Hs.497353 | MED6 | Mediator complex subunit 6 |
| 58 | Hs.517307 | MX1 | Myxovirus resistance 1, interferon-inducible protein p78 |
| 59 | Hs.483564 | PFDN1 | Prefoldin subunit 1 |
| 60 | Hs.497581 | EIF2D | Eukaryotic translation initiation factor 2D |
| 61 | Hs.172865 | CSTF1 | Cleavage stimulation factor, 3' pre-RNA, subunit 1, 50kDa |
| 62 | Hs.465305 | TMEM51 | Transmembrane protein 51 |
| 63 | Hs.353175 | AGPAT4 | 1-acylglycerol-3-phosphate O-acyltransferase 4 |
